# Quantification and reduction of peripheral dose from leakage radiation on Siemens primus accelerators in electron therapy mode

**DOI:** 10.1120/jacmp.v11i3.3105

**Published:** 2010-06-15

**Authors:** Collins Yeboah, Alex Karotki, Dylan Hunt, Rick Holly

**Affiliations:** ^1^ Department of Medical Physics Odette Cancer Centre Toronto Ontario; ^2^ Department of Radiation Oncology University of Toronto Toronto Ontario

**Keywords:** electron beams, leakage radiation, electron applicator leakage, peripheral dose

## Abstract

In this work, leakage radiation from EA200 series electron applicators on Siemens Primus accelerators is quantified, and its penetration ability in water and/or the shielding material Xenolite‐NL established. Initially, measurement of leakage from $10\times 10-25\times 25\;{\text{cm}}^{2}$ applicators was performed as a function of height along applicator and of lateral distance from applicator body. Relative to central‐axis ionization maximum in solid water, the maximum leakage in air observed with a cylindrical ion chamber with 1 cm solid water buildup cap at a lateral distance of 2 cm from the front and right sidewalls of applicators were 17% and 14%, respectively; these maxima were recorded for 18 MeV electron beams and applicator sizes of $\geq 20\times 20\;{\text{cm}}^{2}$. In the patient plane, the applicator leakage gave rise to a broad peripheral dose off‐axis distance peak that shifted closer to the field edge as the electron energy increases. The maximum peripheral dose from normally incident primary electron beams at a depth of 1 cm in a water phantom was observed to be equal to 5% of the central‐axis dose maximum and as high as 9% for obliquely incident beams with angles of obliquity ≤ 40°. Measured depth‐peripheral dose curves showed that the “practical range” of the leakage electrons in water varies from approximately 1.4 to 5.7 cm as the primary electron beam energy is raised from 6 to 18 MeV. Next, transmission measurements of leakage radiation through the shielding material Xenolite‐NL showed a 4 mm thick sheet of this material is required to attenuate the leakage from 9 MeV beams by two‐thirds, and that for every additional 3 MeV increase in the primary electron beam energy, an additional Xenolite‐NL thickness of roughly 2 mm is needed to achieve the aforementioned attenuation level. Finally, attachment of a 1 mm thick sheet of lead to the outer surface of applicator sidewalls resulted in a reduction of the peripheral dose by up to 80% and 74% for 9 and 18 MeV beams, respectively. This sidewall modification had an insignificant effect on the clinical depth dose, cross‐axis beam profiles, and output factors.

PACS numbers: 87.53.Bn, 87.56.bd, 87.56.J‐

## I. INTRODUCTION

In electron beam therapy, cones or applicators are utilized in conjunction with cerrobend cutouts for defining the radiation field size and shape in the vicinity of the patient plane. The rationale for using cones/applicators is to try to confine the electron beam by minimizing the lateral spread of the beam that occurs in the intervening air volume from inside the accelerator head to the patient surface. The efficacy of an applicator design lies in its ability to block scattered electrons generated in the machine head and/or air gap from escaping through the applicator sidewalls and becoming leakage radiation. Previous studies have shown that the leakage radiation on the outside surface of an applicator sidewall or just outside the applicator body can be prohibitively high.^(^ ^1^ ^–^ ^5^ ^)^ This, in turn, leads to unacceptably high peripheral doses in the patient plane^(^ ^1^ ^–^ ^3^ ^)^ and can be detrimental to the patient, especially when sensitive structures and/or the patient's skin is in close proximity to the applicator sidewall – as in the treatment of breast cancer involving the internal mammary lymph node chain (IMC) using a combination of parallel‐opposed photon tangents and an oblique “electron patch”.^(^ ^6^ ^,^ ^7^ ^)^ Other clinical situations where the applicator leakage poses a significant risk to patients include exposure of the shoulders to leakage radiation when treating neck nodes at extended SSD, and exposure of the legs to leakage during testicular boosts for TBI.^(^ ^8^ ^,^ ^9^ ^)^ In addition, it is well known that exposure of large volumes of normal tissues to low doses of radiation increases an individual's risk of developing a radiation‐induced secondary cancer. Moreover, for patients undergoing electron beam therapy who have implanted cardiac pacemakers and defibrillators, excessive leakage radiation can contribute significantly to the dose absorbed by these devices and, as a result, can adversely affect their function.^(^ ^10^ ^,^ ^11^ ^)^ It is therefore imperative that the leakage radiation escaping through the applicator sidewalls be kept as low as possible. The goal of this work was to characterize the leakage radiation from the EA200 series electron applicators on Siemens Primus accelerators, quantify the peripheral dose due to the leakage radiation in the patient plane, and find strategies for minimizing the leakage radiation, such as ascertaining whether placement of bolus and/or an attenuating material outside the field on a patient's skin (e.g. over the contra‐lateral breast in IMC treatments) reduces the dose to the skin from leakage radiation. This article is organized as follows: It starts with a brief description of the EA200 series electron applicators used with Siemens primus accelerators. This is followed by a description of the methodology used to measure both the leakage radiation in the applicator plane and the peripheral dose due to leakage radiation in the patient plane. Next, the results of measurements of leakage radiation in the vicinity of applicator body for various combinations of electron energies and applicator sizes are presented and discussed. This is followed by a presentation and discussion of peripheral doses measured in the patient plane as a function of off‐axis distance from field edge, angle of obliquity of incident beams, and depth in a water phantom. In addition, results of transmission measurements of leakage radiation through an attenuating material in the patient plane are presented. Finally, an investigation was conducted to ascertain whether or not the applicator leakage and/or the resulting peripheral dose to a patient could be reduced by modification of applicator sidewalls.

## II. MATERIALS AND METHODS

### A. Description of a fixed electron applicator

**Figure 1:**
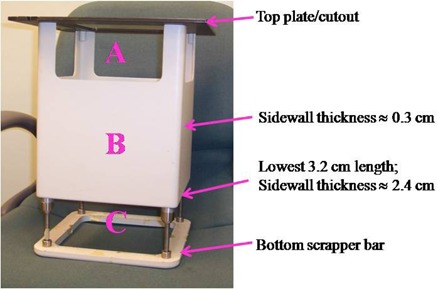
An EA220 ($20\times 20\;{\text{cm}}^{2}$) electron applicator used with Siemens Primus accelerators.

A dual modality Siemens Primus unit with four EA200 series electron applicators of fixed sizes 10 × 10, 15 × 15, 20 × 20 and $25\times 25\;{\text{cm}}^{2}$ at the isocenter was employed for this work. Each applicator consists of a top plate (i.e. a field‐defining cut‐out) that fits into the accessory tray holder of the treatment head, four sidewalls, and a bottom scrapper bar (Fig. 1). Each sidewall is made up of three main regions: (i) region A is the area at the top with no physical wall and runs from the top plate/cutout of applicator down to a length of 9 cm; (ii) region B is the middle half of the sidewall that is composed of a real physical wall of aluminum and has a length of 19.4 cm; (iii) the remainder of the sidewall – that is, the bottom 8.6 cm length of it – is designated as region C and it is wall‐less. With the exception of the lowest 3 cm length of region B where the sidewall is 2.1–3.2 cm thick, each sidewall constituting region B has a uniform thickness of between 0.3 and 0.6 cm, depending on the size of applicator under consideration (See Table 1). For example, in the case of the $20\times 20\;{\text{cm}}^{2}$ applicator shown in Fig. 1, the sidewall has a uniform thickness of 0.3 cm except for the lowest 3.2 cm length of it where the sidewall is thicker by 2 cm. Also, different sized applicators employ different X‐ray jaw settings ranging from 19 × 19 to $32\times 32\;{\text{cm}}^{2}$ for applicator sizes of 10 × 10 to $25\times 25\;{\text{cm}}^{2}$, as shown in Table 1. For a given applicator, the corresponding X‐ray jaw settings are the same for all incident electron energies. Unless otherwise specified, when the gantry and collimator angles are both set to zero, the front/back side of an applicator designates the X‐ray target/gun side, whereas the right side of an applicator refers to the right‐hand side of an observer standing in front of the gantry and facing it.

**Table 1:** Some characteristics of EA200 series electron applicators used with Siemens Primus accelerators.

*Applicator size (cm^2^)*	*Manufacturer code*	*X‐Ray jaw settings (cm^2^)*	*Applicator sidewall thickness (cm)*	*“Mini‐phantom‐in‐sidewall” thickness* a *(cm)*	*Original weight of applicator (kg)*	*Modified weight of applicator (kg)*
10 × 10	EA210	19 × 19	0.65	3.1	7.4	8.7
15 × 15	EA215	23 × 23	0.32	2.8	7.6	9.5
20 × 20	EA220	27 × 27	0.33	2.4	8.2	10.4
25 × 25	EA225	32 × 32	0.33	2.1	8.7	11.3

a In region B of applicator sidewall, the lowest 3 cm length of the sidewall is thicker and thus may be considered as a “mini‐phantom” in the sidewall.

### B. Measurement of leakage radiation through fixed electron applicators

**Figure 2:**
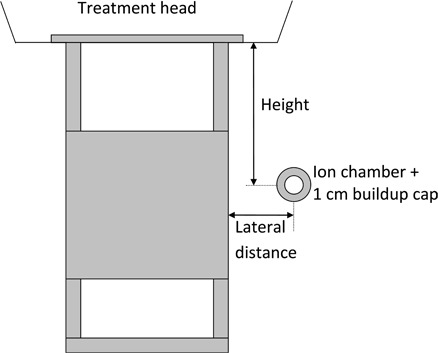
Schematic of the setup employed for measuring leakage radiation in the vicinity of applicator body.

Measurement of leakage radiation through fixed electron applicators on Siemens Primus units was performed using a Farmer chamber (Model PR‐06G, Capintec Inc., Ramsey, NJ, USA) with a 1 cm “virtual water” build‐up cap. Although the central‐axis dose maximum for 6, 9, 12, 15 and 18 MeV incident primary electron beams occur at depths of 1.4, 2.2, 2.8, 2.0, and 2.0 cm, respectively, “depth‐peripheral dose” curves measured in water for the leakage electrons showed the peripheral dose maximum for each energy occur close to the surface of the phantom. Thus, the use of a build‐up cap thickness of 1 cm for all energies was appropriate for these measurements. In all cases, both the gantry and collimator angles were set to zero. As demonstrated in Fig. 2, the detector – that is, ion chamber plus 1 cm build‐up cap – was positioned in air with axis of detector at a distance of 2 cm from applicator body and oriented such that its axis was parallel to the gun‐target direction for measurements on the right side of applicator. For measurements on the front side of applicator, the detector axis was made perpendicular to the gun‐target direction. First, with the detector positioned on the right side of applicator at a distance of 2 cm from its sidewall, measurements of leakage were made as a function of height along applicator body for each incident electron energy (6–18 MeV) and applicator size. From these data, the maximum leakage and its location on the right side of applicator body was recorded. The above procedure was repeated on the front side of applicator. Second, with the detector positioned at the location of maximum leakage on the front side of applicator, measurements of leakage were performed as a function of lateral distance from applicator body. Unless otherwise specified, the leakage measured for a given electron energy‐applicator size combination refers to the ionization measured in the vicinity of applicator sidewall and normalized to the corresponding central‐axis ionization maximum in a solid water phantom.

### C. Measurement of peripheral dose in the patient plane

#### C.1 Dependence of peripheral dose on off‐axis distance

To assess the contribution of applicator leakage radiation to peripheral doses in the patient plane, the peripheral dose at a depth of 1 cm in a water phantom was measured as a function of off‐axis distance from field edge using an electron diode detector. The measurements were performed from the field edge out to a distance of 40 cm from the edge for 10 × 10 and $20\times 20\;{\text{cm}}^{2}$ open applicators and 6–18 MeV electron beams using a source‐to‐surface distance of 100 cm. Unless otherwise stated, the doses measured at off‐axis points of interest are normalized to the central‐axis dose maximum in a water phantom for the given electron energy.

#### C.2 Peripheral dose versus angle of beam obliquity

**Figure 3:**
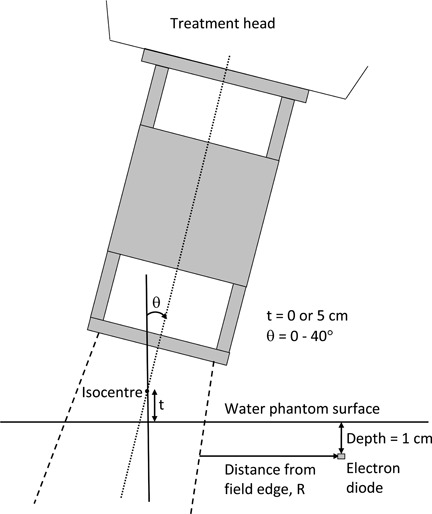
Schematic of the experimental setup used for measuring peripheral doses. Measurements performed as a function of angle of beam obliquity, Θ, had isocenter‐to‐phantom surface distance, t, held constant at 5 cm.

To ascertain the effect of oblique beam incidence on peripheral dose due to leakage radiation, peripheral doses measured with the beam at normal incidence on the water phantom surface were compared to those measured when the beam was incident obliquely on the phantom surface. Using the setup illustrated in Fig. 3, two experiments were performed with an electron diode detector positioned at a depth of 1 cm in water: (i) using the $20\times 20\;{\text{cm}}^{2}$ applicator, off‐axis peripheral dose profiles for a normally incident beam and a beam incident obliquely at 40° on the phantom surface were compared for 9 and 18 MeV electron beams; (ii) with the detector positioned at 1 cm depth in water in the vicinity of the off‐axis point at which the peripheral dose‐distance peak occurs (14 cm from the field edge for electron energies ≤ 12 MeV and 10 cm for beam energies ≥ 15 MeV), peripheral dose was measured for each applicator as a function of beam obliquity for 6–18 MeV incident electron beams. This was accomplished by varying the gantry angle from 0°, which corresponds to normal incidence on the phantom surface, to 40° and in each case repositioning the detector laterally such that it remains at the same distance from the field edge for all measurements (Fig. 3). An oblique angle of 40° was selected to simulate IMC treatments using parallel‐opposed photon tangents and an oblique electron patch. Typically, the electron beam used for such treatments are incident at gantry angles of less than 40°. Thus, the selection of this oblique angle was intended to mimic a worst‐case scenario. In all cases, the depth of measurement was 1 cm and was measured along an axis perpendicular to the phantom surface.

#### C.3 Penetration ability of leakage radiation in a water phantom

The penetration ability in water of applicator leakage radiation was determined using a water phantom and an electron diode detector. The phantom was positioned in the patient plane at a source‐to‐surface plane distance of 100 cm at the front side of a $20\times 20\;{\text{cm}}^{2}$ applicator such that the lateral distance of the detector from the field edge was 12 cm. Thus, the distance from the bottom of applicator to the phantom surface was 5 cm. The gantry and collimator angles were both set to 0°, and measurement of depth‐dose on the front side of the applicator along an axis parallel to the central‐axis but located at a distance of 12 cm from the field edge was performed. The measured data for a given electron energy was normalized to the corresponding central‐axis dose maximum in the water phantom. The measurement of depth‐dose was repeated using the $10\times 10\;{\text{cm}}^{2}$ applicator.

### D. Transmission of applicator leakage radiation through Xenolite‐NL

The transmission studies of applicator leakage radiation employed the radiation shielding material Xenolite‐NL (Lite Tech, Inc., Norristown, PA 19403, USA). This material is lead‐free and is composed of tungsten and antimony in a plasticized elastomeric matrix. It was chosen for this work because it is free of lead, flexible, and can be easily cut to any desired size. The thicknesses of Xenolite‐NL needed to adequately shield the electron applicator leakage in the patient plane were determined using a thin‐window parallel plate ion chamber (Markus model N23343, PTW, Freiburg, Germany) and the $20\times 20\;{\text{cm}}^{2}$ applicator. The chamber was positioned at a depth of 0.2 cm in a solid water phantom of size $30\times 30\times 30\;{\text{cm}}^{3}$, whose surface plane was perpendicular to the central axis of the primary electron beam. The source‐to‐surface plane distance measured along the central axis was kept constant at 100 cm for all measurements, while the gantry and collimator angles were both set to 0. To make transmission measurements of leakage radiation at the front side of applicator, the phantom was displaced laterally away from the central axis in the “in‐plane” direction such that the detector was located at a lateral distance of 22 cm from the central axis. Using sheets of Xenolite‐NL each of surface area $15\times 15\;{\text{cm}}^{2}$ and thicknesses ranging from 0 to 10 mm as attenuating material placed on the surface of the solid water phantom, the leakage transmitted through this material was measured at 0.2 cm depth of solid water for 6, 9, 12, 15 and 18 MeV incident electron beams. The ionization measured with a given thickness of Xenolite‐NL in place was normalized to (i) the ionization measured at the depth of maximum dose on the central axis in a solid water phantom, and (ii) the ionization observed at the 0.2 cm solid water depth in the absence of the Xenolite‐NL attenuating material.

### E. Reduction of applicator leakage radiation

To reduce the leakage radiation escaping through the applicator sidewalls, each applicator (10 × 10 to $25\times 25\;{\text{cm}}^{2}$) was modified by wrapping a sheet of lead of thickness 1 mm around the outer surface of the sidewalls such that the sheet covered only the sidewalls' middle half that already consists of a physical wall (see region B in Fig. 1). The rationale for utilizing a lead thickness of 1 mm was not only based on weight considerations but also to minimize any potential influence on the clinical electron beams by the lead sheet's attachment.^(^ ^1^ ^)^ In Table 1, the weights of the original and modified applicators are shown. The effect of applicator sidewall modification on peripheral dose was assessed by comparing peripheral doses measured in a water phantom with and without the 1 mm thick lead sheet in place. First, peripheral dose off‐axis distance curves at a depth of 1 cm in water were measured on the right side of applicator with gantry and collimator angles of 40° and 0°, respectively. Second, depth‐peripheral dose curves were acquired at the front side of applicator using a gantry angle of 40° and a collimator angle equal to 90°. [Note that the front side of applicator for a collimator angle of 90° corresponds to the left side of a patient in supine position with head pointing towards the gantry]. In this case, depth was measured along an axis perpendicular to the phantom surface and intersecting the surface at a lateral distance of 10 cm from the field edge (Fig. 3). In addition, to ascertain the influence of the lead sheet's attachment to applicator sidewalls on the clinical beams, central‐axis depth doses, cross‐beam profiles, and output factors measured before and after the sidewall modification were compared for 10 × 10 and $20\times 20\;{\text{cm}}^{2}$ applicators.

## III. RESULTS & DISCUSSION

### A. Dependence of leakage on vertical position along applicator

**Figure 4:**
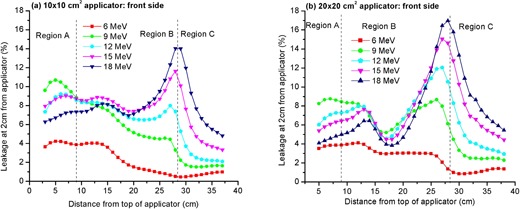
Leakage at a lateral distance of 2 cm from applicator body (relative to central‐axis ionization maximum in a water‐equivalent phantom) as a function of vertical position along applicator: (a) front side of $10\times 10\;{\text{cm}}^{2}$ applicator; (b) front side of $20\times 20\;{\text{cm}}^{2}$ applicator.

Figures 4(a) and 4(b) depict the leakage at “1 cm depth” of solid water measured at a lateral distance of 2 cm from the front side of applicator body as a function of vertical position along applicator for the 10 × 10 and $20\times 20\;{\text{cm}}^{2}$ applicators, respectively. As the incident electron beam energy increases, the leakage detected around the top region of applicator sidewall (region A, comprising the top 9 cm length) increases to a maximum at 9 MeV and thereafter decreases with increasing incident energy (Fig 4(a‐b)). This is because the low energy electron beams exhibit more lateral spread in the treatment head than the high energy beams due to multiple Coulomb scattering in the head and intervening air. Consequently, on emerging from the treatment head the low energy electron beams will have more angular spread and a correspondingly higher leakage outside the top region (region A) of applicator body.

In contrast, leakage monotonically increases with incident electron beam energy in the region surrounding the lower half of applicator body. This happens because the middle‐half of an applicator sidewall (region B) is made up of an attenuating material composed of aluminum whose thickness is uniform throughout this region except for the lowest 3 cm length of it, where the sidewall is thicker by 1.8–2.5 cm, depending on the size of applicator under consideration. This “thick‐walled” strip of sidewall acts as a “mini‐phantom” and, as a result, generates significantly more scattering in it than the rest of region “B”. The higher the energy of the incident electron beam, the larger the range of electrons generated in the mini‐phantom. Thus, more leakage radiation will originate from the thick‐walled strip of region “B” for high energy than for low energy electron beams. It follows that leakage radiation in the region surrounding the lower half of applicator body will increase with incident electron energy.

In Table 2, the maximum leakage observed around each applicator (10 × 10 to $25\times 25\;{\text{cm}}^{2}$) for a number of incident electron beam energies is presented. In general, the maximum leakage at the front side of an applicator was observed to be higher than that at the right side for a given incident electron energy. The highest leakage recorded for the front and right sides of applicators were 17% and 14%, respectively, relative to the central‐axis ionization maximum in a solid water phantom; these maxima occurring for 18 MeV electron beams and applicator sizes of $\geq 20\times 20\;{\text{cm}}^{2}$ exceed the IEC recommended limit of 10%.^(^ ^12^ ^)^ In general, the leakage maximum tended to increase with incident electron beam energy.

**Table 2:** Maximum leakage measured in air with a cylindrical ion chamber using a 1 cm solid water buildup cap at a lateral distance of 2 cm from applicator body relative to the central‐axis dose maximum in a solid water phantom at 100 cm SSD.

	*Maximum leakage at 2 cm from applicator body (%)*							
	$10\times 10\;cm^{2}$ *Appl.*		$15\times 15\;cm^{2}$ *Appl.*		$20\times 20\;cm^{2}$ *Appl.*		$25\times 25\;cm^{2}$ *Appl.*	
*Energy (MeV)*	*Front*	*Right*	*Front*	*Right*	*Front*	*Right*	*Front*	*Right*
6	4.0	2.7	4.6	3.4	4.0	2.7	4.3	2.8
9	8.4	5.0	12.9	8.5	8.7	7.4	11.9	6.7
12	8.4	4.7	13.0	8.5	12.0	10.1	13.0	9.4
15	11.6	7.2	14.2	8.4	14.9	12.5	14.1	13.0
18	14.1	9.4	13.3	9.4	16.8	13.7	16.0	14.0

### B. Dependence of leakage on lateral distance from applicator body

**Figure 5:**
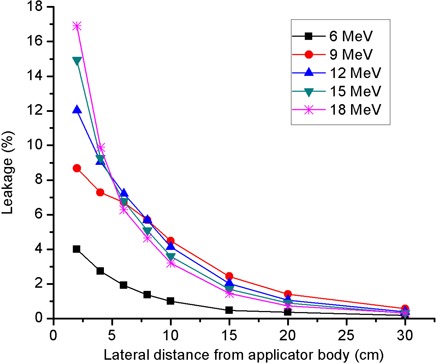
Dependence of leakage in the vicinity of applicator sidewall on lateral distance from applicator body. The presented data, acquired at the front side of a $20\times 20\;{\text{cm}}^{2}$ applicator, are normalized to the central‐axis ionization maximum in a water‐equivalent phantom.

In Fig. 5, the results of leakage measurements at the front side of the $20\times 20\;{\text{cm}}^{2}$ applicator as a function of lateral distance from applicator body are presented. As expected, the leakage decreases with increasing lateral distance from the applicator body. At a lateral distance of 2 cm from applicator body, the observed maximum leakage was as high as 17% relative to the central‐axis ionization maximum. However, at lateral distances of ≥4cm from the applicator body, the maximum leakage recorded was less than 10% for each incident energy and applicator size combination, whereas at lateral distances of >10cm, the maximum leakage was observed to be ≤5% of the central‐axis ionization maximum in all cases. The 6 MeV curve appears different because the range of its leakage electrons in water‐equivalent material is so limited that only a small fraction of them are able to traverse the 1 cm build‐up cap of the ion chamber and be detected. This is, however, not the case for the higher energy beams whose leakage electrons have sufficient energies to enable them to traverse the 1 cm build‐up cap and be detected.

### C. Dependence of peripheral dose in the patient plane on off‐axis distance

**Figure 6:**
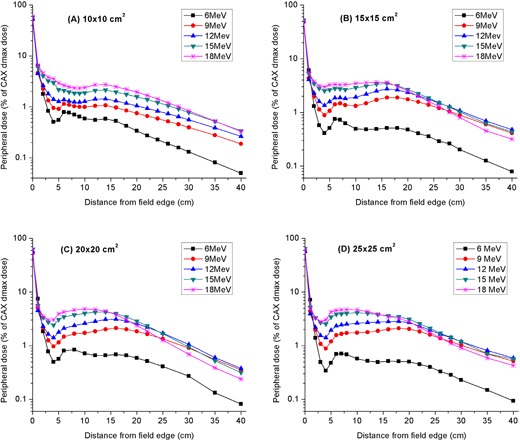
Dependence of peripheral dose in the patient plane on off‐axis distance from the field edge for (a) 10 × 10, (b) 15 × 15, (c) 20 × 20, and (d) $25\times 25\;{\text{cm}}^{2}$ applicators. Measurements were performed at a depth of 1 cm in a water phantom, at 100 cm SSD.

Figure 6 depicts the off‐axis peripheral dose profile as a function of lateral distance from field edge for the 10 × 10 to $25\times 25\;{\text{cm}}^{2}$ applicators. As the distance from the field edge increases, the peripheral dose decreases to a minimum, but afterwards increase to form a broad peak of up to 5% of central‐axis dose maximum. The peripheral dose eventually starts to decrease again with further increase in lateral distance. The magnitude and location of the peak peripheral dose depend on the incident electron energy and applicator size employed. First, the peak peripheral dose increases with incident electron beam energy. This is because a progressively greater amount of leakage radiation escapes through the sidewalls of the applicators as the incident electron beam energy becomes higher. Second, for the $10\times 10\;{\text{cm}}^{2}$ applicator, the peripheral dose peaked at approximately the same distance of 14 cm from the field edge irrespective of incident beam energy. However, as the size of the applicator increases, the location of the peripheral dose peak shifts farther away from the field edge, reaching a distance of 19 cm for the low energy (≤ 12 MeV) electron beams. In contrast, for the high energy (≥ 15 MeV) beams, the peripheral dose peak moves towards the field edge as the size of applicator increases.

### D. Peripheral dose versus angle of beam obliquity

**Figure 7:**
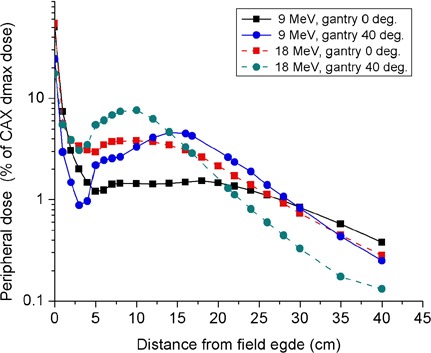
Dependence of peripheral dose on angle of beam obliquity for $20\times 20\;{\text{cm}}^{2}$ applicator. Measurement was performed at the right side of applicator at a depth of 1 cm in water using isocenter‐to‐phantom surface distance, t=5 cm. Data obtained for normal beam incidence are compared to those acquired for a 40° obliquely incident beam.

**Figure 8:**
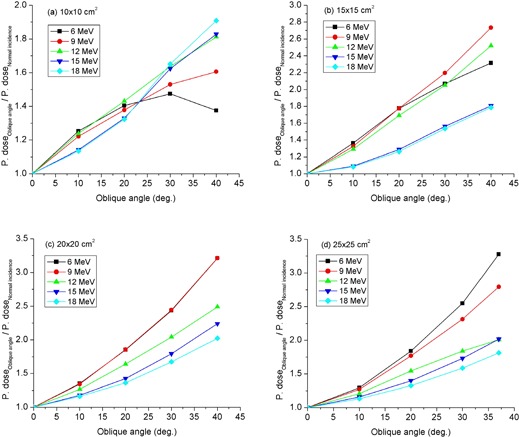
Dependence of peripheral dose on angle of beam obliquity for (a) $10\times 10\;{\text{cm}}^{2}$, (b) $15\times 15\;{\text{cm}}^{2}$, (c) $20\times 20\;{\text{cm}}^{2}$, and (d) $25\times 25\;{\text{cm}}^{2}$ applicators. Using a constant isocenter‐to‐phantom surface distance, t=5 cm, measurements were performed at 1 cm depth in water at off‐axis distances of 14 cm and 10 cm from the field edge for electron beam energies of ≤ 12 MeV and ≥ 15 MeV, respectively.

The effect of oblique beam incidence on peripheral dose at 1 cm depth in water is demonstrated in Fig. 7, in which peripheral dose profiles for normally incident and 40° obliquely incident beams with respect to the phantom surface are compared for 9 and 18 MeV electron beams. The data were acquired in the patient plane at the right side of $20\times 20\;{\text{cm}}^{2}$ applicator using an SSD of 105 cm. One notes that the peripheral dose peak increases sharply and shifts towards the field edge as the angle of obliquity increases. In these cases, the peak peripheral dose increased by factors of approximately 3 and 2 for incident electron beams of energies 9 and 18 MeV, respectively. In Fig. 8, the angle of obliquity dependence of peripheral dose at a specified off‐axis distance from the field edge is presented for a number of applicator sizes. These data were measured at a lateral distance of 14 cm from the field edge for 6–12 MeV beams and at 10 cm for beam energies of 15 MeV and higher. One notes that the peripheral dose from obliquely incident beams increases sharply with angle of beam obliquity on phantom surface. Relative to measurements performed at normal beam incidence, peripheral dose from obliquely incident beams of angles ≤ 40° are up to three times higher for low energy beams and up to twice as high for high energy beams. In other words, for 9 and 18 MeV beams incident on phantom surface at an oblique angle of 40° relative to normal beam incidence, the peripheral dose maxima observed are as high as 7% and 10%, respectively. These have implications for treatment of patients implanted with cardiac pacemakers and defibrillators.

### E. Penetration ability of applicator leakage in a water medium

**Figure 9:**
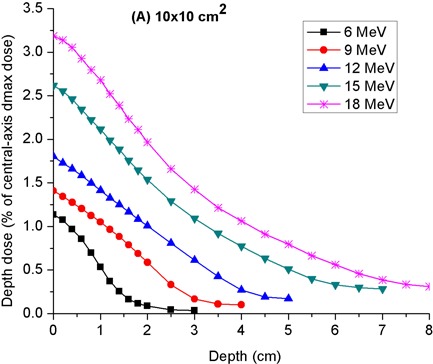
Depth‐peripheral dose curves in water due to leakage radiation from $10\times 10\;{\text{cm}}^{2}$ applicator. These data were acquired at the front side of applicator, in the patient plane, along an axis parallel to the central axis but located at a lateral distance of 17 cm from it.

Figure 9 depicts the off‐axis depth‐dose curves measured at a lateral distance of 12 cm from the field edge for the 10 × 10 and $20\times 20\;{\text{cm}}^{2}$ applicators. Each curve is normalized to the central‐axis dose maximum in a water phantom for the energy in question. Interestingly, dose maxima occur at the phantom surface and one does not see the characteristic initial buildup of dose at shallow depths. This is due to the fact that the applicator leakage electrons incident on the phantom surface are highly diffused. As a result, they do undergo only a modest additional angular spread upon entering the phantom. Thus, the initial buildup of electron fluence associated with increased angular spread of electrons in a phantom is absent. As expected, the relative depth‐dose increases with incident electron beam energy. For a given electron energy, the relative depth‐dose curve of the $10\times 10\;{\text{cm}}^{2}$ applicator is lower and also falls off more rapidly at shallow depths than that of the $20\times 20\;{\text{cm}}^{2}$ applicator. In fact, the shape of the curves for the $20\times 20\;{\text{cm}}^{2}$ applicator is similar to typical central‐axis depth‐dose curves, save for the absence of an initial dose buildup. As a result, the most probable energy of the applicator leakage electrons on the phantom surface was inferred from a determination of the “practical range” and application of TG‐25^(^ ^13^ ^)^ methodology (see Table 3). The results show that the most probable energy of the electron leakage on the surface of the water phantom varies from approximately 3.0 to 11.8 MeV as the primary electron energy is raised from 6 to 18 MeV. The application of the above methodology to determine the incident energy of the leakage electrons is only an approximation since the relationship between the practical range and incident energy is applicable for fairly monoenergetic beams under well‐defined geometry.

**Table 3:** The most probable energy of leakage electrons from $20\times 20\;{\text{cm}}^{2}$ applicator determined at the surface of a water phantom using the methodology of TG‐25.^(^ ^13^ ^)^

*Nominal Energy Primary Electrons*, $E_{\mathrm{nom}}$ *(MeV)*	*Practical Range Leakage*, $R_{p,Leakage}(cm)$	*Most Probable Energy Leakage*, $R_{p0,Leakage}(MeV)$	*Leakage‐to‐Primary Energy Ratio*, $E_{p0,Leakage,0}/E_{nom}$
6	1.4	3.0	0.50
9	2.5	5.2	0.58
12	3.3	6.9	0.57
15	4.5	9.3	0.62
18	5.7	11.8	0.66

At depths of <2 cm of water, the peripheral dose falls to 1%–2% of central‐axis dose maximum for energies of 12 MeV and lower. Thus, it is feasible to use 1.5 and 2 cm thick bolus material placed on a patient's skin to shield the applicator leakage radiation for 9 and 12 MeV electron beams, respectively. However, since the depth‐peripheral dose for higher energy beams falls to 1%–2% at depths of at least 3–4 cm, it is not practical to use bolus of similar thicknesses for shielding the applicator leakage radiation from electron beams of energies ≥ 15 MeV.

### F. Transmission of applicator leakage through Xenolite‐NL

**Figure 10:**
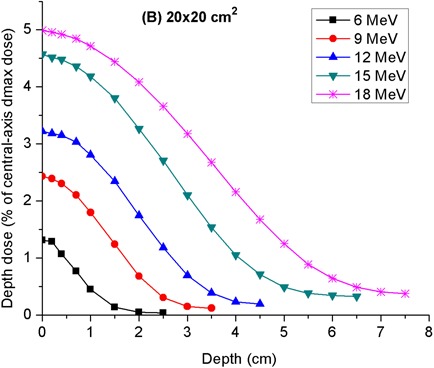
Depth‐peripheral dose curves in water due to leakage radiation from $20\times 20\;{\text{cm}}^{2}$ applicator. These data were acquired at the front side of applicator, in the patient plane, along an axis parallel to the central axis but located at a lateral distance of 22 cm from it.

Transmission measurements in the patient plane of leakage radiation from a $20\times 20\;{\text{cm}}^{2}$ applicator through Xenolite‐NL are presented in Fig. 10(a) for incident electron beams of energies 6–18 MeV. The presented data in Fig. 10(a) depicts the ratio of ionization collected at the 0.2 cm solid water depth with a given thickness of Xenolite‐NL in place to that measured at the depth of maximum dose on the central axis in a solid water phantom. As the thickness of Xenolite‐NL increases, the transmitted leakage builds up to a maximum and thereafter decreases. This initial buildup is less pronounced for the low energy beams. As expected, the leakage radiation becomes more penetrating as the primary electron beam energy increases. In Fig. 10(b), the reported transmission represents the ratio of ionization collected at the 0.2 cm solid water depth when a specified thickness of Xenolite‐NL was used to that measured at the same point in the absence of the Xenolite‐NL attenuating material. These transmission data may be utilized to select an appropriate thickness of Xenolite‐NL for shielding the leakage radiation on a patient's skin. In doing so, one should keep in mind that the peripheral dose and, hence, the required thickness of shielding material depends on a number of factors including: (i) primary electron beam energy, (ii) applicator size, (iii) off‐axis distance from field edge, and (iv) angle of beam obliquity with respect to the patient's surface. The selected thickness of Xenolite‐NL should be such that it reduces the peripheral‐dose maximum for a given electron energy‐applicator size combination to ≤3%, say, of the central‐axis dose maximum in a water phantom. Since in a worse‐case scenario of using a large‐sized applicator ($\geq 20\times 20\;{\text{cm}}^{2}$) and an obliquely incident beam with angle of obliquity Θ≤ 40° the peripheral dose‐maxima are approximately 6% and 9% relative to central‐axis dose maximum in water for 9 and 18 MeV incident beams, respectively (refer to Fig. 7), it follows that the selected thickness of Xenolite‐NL in this case should be such that it reduces the peripheral dose by roughly two‐thirds or 65%–70%. According to Fig. 10(b), the thicknesses of Xenolite‐NL required to attenuate the leakage radiation in the patient plane by 65%–70% varies from approximately 4 to 10 mm as the primary electron beam energy is raised from 9 to 18 MeV. That is, for every 3 MeV increase in primary electron beam energy above 6 MeV, an additional Xenolite‐NL thickness of roughly 2 mm is required to achieve the aforementioned attenuation level. At each field edge of interest, one may place the sheets of Xenolite‐NL on the patient's skin from a distance of roughly 2 cm from the outer edge of bottom applicator scrapper bar or its projection on skin to a distance of 22 cm from the edge, to cover an area of length approximately 20 cm (refer to Fig. 6). The width of the Xenolite‐NL sheets should be made at least equal to the width of the applicator in question.

**Figure 11:**
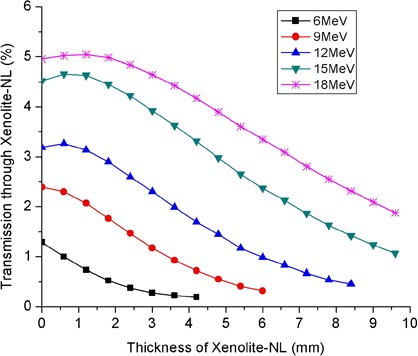
Transmission of applicator leakage radiation from $20\times 20\;{\text{cm}}^{2}$ applicator through Xenolite‐NL for normally incident primary electron beams. The presented data are expressed as percentages of central‐axis “dose” maxima in solid water. The measurements were performed in solid water at depth of 0.2 cm and at a lateral distance of 22 cm from the central axis.

**Figure 12:**
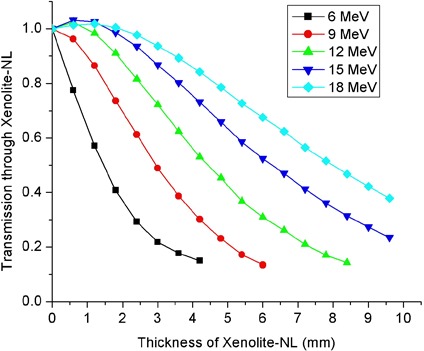
Ratio of transmitted leakage from $20\times 20\;{\text{cm}}^{2}$ applicator through a given thickness of Xenolite‐NL, measured at a depth of 0.2 cm of solid water, to the leakage at the same depth of solid water in the absence of Xenolite‐NL attenuating material.

### G. Reduction of applicator leakage radiation

Figure 11 depicts the peripheral dose‐versus‐lateral distance curves for the standard and lead‐wrapped $20\times 20\;{\text{cm}}^{2}$ applicators. The sidewall modification with the 1 mm thick lead sheet resulted in a reduction in peak peripheral dose of up to 80% and 74% for 9 and 18 MeV incident electron beams, respectively. In Fig. 12, the depth‐peripheral dose curves for the $20\times 20\;{\text{cm}}^{2}$ applicator measured in the absence and presence of the lead sheet attachment to the applicator sidewalls are shown for 9 and 18 MeV incident beams. At the surface of the phantom, the applicator sidewall modification led to a reduction in peripheral dose of 51% and 60% for 9 and 18 MeV beams, respectively. At a depth of 1 cm in the water phantom, the reduction in peripheral dose for 9 and 18 MeV beams resulting from the sidewall modification are 65% and 67%, respectively.

**Figure 13:**
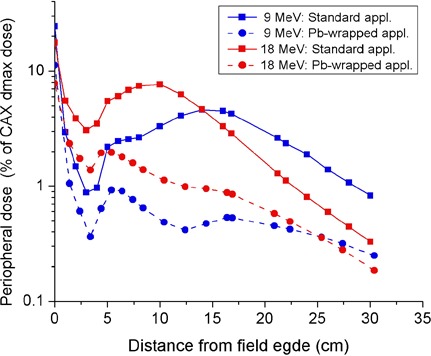
Comparison of peripheral‐dose distance curves measured at a depth of 1 cm in water on the right side of modified sidewall and standard $20\times 20\;{\text{cm}}^{2}$ applicators. An oblique incident angle of 40° and a collimator angle of 0° were employed.

**Figure 14:**
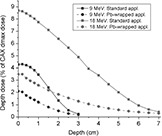
Depth‐peripheral doses measured on the front side of modified sidewall and standard $20\times 20\;{\text{cm}}^{2}$ applicators. The depth was measured along an axis perpendicular to the phantom surface and intersecting the surface at a lateral distance of 10 cm from the field edge. The setup utilized a gantry angle of 40° and a collimator angle of 90°.

On examining the influence of applicator sidewall's modification on the clinical electron beams of energies 6–18 MeV, the following observations were made for the 10 × 10 and $20\times 20\;{\text{cm}}^{2}$ applicators: (i) negligible differences of < 0.5% were seen in the central‐axis depth‐dose curves acquired in the absence and presence of the lead sheet attachment; (ii) cross‐beam profiles measured with and without the applicator sidewall modification had insignificant differences (<1%) between them; (iii) relative output factors measured with the modified sidewall applicators, fitted with and without field‐defining cerrobend cutouts, were within 1% of those measured using the original, unmodified applicators.

### H. Clinical considerations

Using the data presented in this study, one can estimate more accurately the leakage radiation dose to normal tissue and/or sensitive structures located outside the field in electron beam therapy. Also, the leakage reduction techniques proposed in this study can be employed to minimize exposure of normal tissues and sensitive structures to leakage radiation under various clinical situations. For example, the leakage radiation dose deposited in implanted cadiac pacemakers and defibrillators during electron beam therapy of a cardiac patient can be significantly reduced if one of the proposed leakage reduction methods is utilized. Suppose a prescribed dose of 5000 cGy is being delivered in IMC “electron patch” treatment to a patient with implanted cadiac defibrillator using 12 MeV electron beams, $20\times 20\;{\text{cm}}^{2}$ applicator, and angle of obliquity of the beam between 30° and 40°. In this case, the defibrillator could receive leakage radiation dose as high as 250 cGy (0.05×5000 cGy) or more – this is much higher than the recommended tolerance dose limit of 100 cGy.^(^ ^11^ ^)^ However, application of the proposed leakage reduction techniques would reduce the peripheral dose deposited in the defibrillator to a value less than the 100 cGy limit. A similar reduction in leakage radiation dose can be achieved for the contra‐lateral breast in IMC electron patch treatment if either of the aforementioned strategies was employed to minimize the leakage. Another example involves the treatment of neck nodes with electron beams, where the close proximity of the patient's shoulder to the applicator sidewall could lead to exposure of the shoulders to high levels of leakage radiation. In this case, the leakage radiation dose delivered to the shoulders could be as high as 10% of the prescribed target dose. However, deployment of either of the leakage reduction techniques proposed in this study could reduce the leakage radiation dose to the shoulders by up to 80%. The same argument can be made in support of the ability of the proposed leakage reduction techniques in protecting a patient's legs from excessive exposure to leakage radiation during the delivery of a testicular boost for TBI.

## IV. CONCLUSIONS

In this work, leakage radiation in the applicator plane and the resulting peripheral dose in the patient plane have been quantified, and strategies for minimizing the leakage in clinical situations are proposed. The results reveal serious deficiencies in the design of the EA200 series electron applicators. The maximum leakage observed at a lateral distance of 2 cm from the applicator body at the front and right sides of applicator were 17% and 14%, respectively, relative to the central‐axis “dose” maximum in a solid water phantom; these maxima were recorded for 18 MeV incident electron beams and applicator sizes of $\geq 20\times 20\;{\text{cm}}^{2}$. These values exceed the IEC recommended limit of 10%.^(^ ^12^ ^)^ The leakage maximum tended to increase with incident electron beam energy. For high energy electrons beams, it also increased as the size of applicator becomes larger.

In the patient plane, the high applicator leakage gave rise to a broad peripheral dose off‐axis distance peak, centered at off‐axis distances of between 8 and 19 cm from the field edge of an open applicator. The maximum peripheral dose observed at a depth of 1 cm in a water phantom was up to 9% of the central‐axis dose maximum in a water phantom, depending on the incident energy‐applicator size combination and the angle of beam obliquity with respect to the phantom surface. In general, the peripheral dose in the patient plane due to applicator leakage increases as (i) the electron energy increases, (ii) the applicator size increases, and (iii) the angle of beam obliquity with respect to the phantom surface increases. These have implications for a number of clinical situations, for example, the contra‐lateral breast in breast cancer treatments involving the IMC, as well as in the treatment of patients implanted with cardiac pacemakers and defibrillators.

Studies conducted to gain an insight into the penetration ability of the leakage electrons showed the most probable energies of leakage electrons on the water phantom surface were approximately 3.0–11.8 MeV for primary electron beams of energies in the range 6–18 MeV.

Transmission studies of applicator leakage radiation through Xenolite‐NL in the patient plane showed that the leakage and hence the peripheral dose to a patient's skin from the leakage radiation can be reduced to at least one‐third of its original value by shielding with 4–10 mm thick piece of Xenolite‐NL when treating with electron beams of energies in the range of 9–18 MeV. Alternatively, attachment of a sheet of lead of thickness 1 mm to the outer surface of applicator sidewalls decreased the peripheral dose in the patient plane by up to 80% and 74% for 9 and 18 MeV beams, respectively. This sidewall modification had an insignificant effect on the clinical beam profiles and output factors.

## ACKNOWLEDGMENTS

We wish to thank Harry Easton and Gerard Peterson for fabricating accessories used in this work.
